# The bright and dark sides of egoism

**DOI:** 10.3389/fpsyt.2022.1054065

**Published:** 2022-11-24

**Authors:** Martin Weiß, Vassil Iotzov, Yuqing Zhou, Grit Hein

**Affiliations:** Translational Social Neuroscience Unit, Department of Psychiatry, Center of Mental Health, Psychosomatic and Psychotherapy, University of Würzburg, Würzburg, Germany

**Keywords:** egoism, incentives, prosociality, social motives, fMRI

## Abstract

Despite its negative reputation, egoism – the excessive concern for one’s own welfare – can incite prosocial behavior. So far, however, egoism-based prosociality has received little attention. Here, we first provide an overview of the conditions under which egoism turns into a prosocial motive, review the benefits and limitations of egoism-based prosociality, and compare them with empathy-driven prosocial behavior. Second, we summarize studies investigating the neural processing of egoism-based prosocial decisions, studies investigating the neural processing of empathy-based prosocial decisions, and the small number of studies that compared the neural processing of prosocial decisions elicited by the different motives. We conclude that there is evidence for differential neural networks involved in egoism and empathy-based prosocial decisions. However, this evidence is not yet conclusive, because it is mainly based on the comparison of different experimental paradigms which may exaggerate or overshadow the effect of the different motivational states. Finally, we propose paradigms and research questions that should be tackled in future research that could help to specify how egoism can be used to enhance other prosocial behavior and motivation, and the how it could be tamed.

## Introduction

“The most disinterested love is, after all, but a kind of bargain, in which the dear love of our own selves always proposes to be the gainer some way or other” (1).

“We have only one relation to each other, that of *usableness*, of utility, of use. We owe *each other* nothing, for what I seem to owe you I owe at most to myself. If I show you a cheery air in order to cheer you likewise, then your cheeriness is of consequence to *me*, and my air serves *my* wish; to a thousand others, whom I do not aim to cheer, I do not show it” (2).

Egoism is commonly related to the “dark side” of human nature, that is, anti-social behavior such as hostility and aggression (3). It is defined as “the excessive concern with one’s own pleasure or advantage at the expense of community well-being” (4). Similarly defined terms that are often synonymous with egoism are selfishness, defined as “an inordinate focus on one’s own welfare, regardless of the well-being of others” (5), and egocentrism, defined as an “excessive interest in oneself and concern for one’s own welfare or advantage at the expense of or in disregard of others” (6). On the motivational level, an egoistic motive drives behavior with the ultimate goal of increasing one’s own welfare (7).

Interestingly, egoism has also been related to behaviors on the “bright side” of human nature such as prosocial behaviors that benefit others than oneself, including a variety of actions like helping, sharing, comforting and cooperating (8). There is evidence that an egoistic motive can drive prosocial behaviors, related to the expectation of a reward. These rewards can be financial incentives, or social rewards such as gain of a positive (i.e., prosocial) reputation, but also by the avoidance of punishment (9). It has been suggested that egoistic motives are promising for promoting the common good because they are easily aroused and are strongly motivating (10).

Another motive for prosocial behavior that is traditionally associated with the “bright side” of human nature is empathy. Empathy is a multidimensional construct (11) with many different definitions (12). Reflecting this multidimensionality, researchers commonly distinguish between affective empathy, i.e., the sharing of others emotions, and cognitive empathy, i.e., the sharing of others cognitive representations (ideas, thoughts, intentions (13–16). It is assumed that both, affective and cognitive empathy can incite prosocial behaviors (13, 17, 18), i.e., behaviors that maximize the outcome of another person at costs to oneself (19, 20). This claim is bolstered by many studies showing a consistent relationship between individual differences in empathy ratings and individual difference in prosocial decisions (19–23). Based on this evidence, an influential social psychology model has proposed that empathy motivates prosocial behavior, because it induces an “altruistic motivation” (24, 25). Building on this model, in this review we refer to empathy as a motive, i.e., a process that can motivate prosocial behavior, in agreement with other researchers of the field (19, 20, 26–28).

Empathy-induced prosocial behavior has been addressed in a number of previous review papers (29–32). In contrast, egoism-based prosociality has received considerably less attention. In the following, we review behavioral and neuroscientific evidence for prosocial behavior that is driven by egoism and discuss its advantages and disadvantages compared to empathy-driven prosocial behavior.

## Why is it important to compare different motives (in the brain)?

In general, behavior is rarely driven by a single motive and multiple motives hardly operate in isolation. There is broad evidence that different motives are often activated simultaneously and most complex behaviors are driven by the interaction between different motives (33). To understand and predict human behavior, it is necessary to investigate the interactions between different motives. However, based on behavioral observations this is challenging, because different motives can result in the identical behavioral outcome. For instance, the same prosocial decision can be driven by an egoistic motive, i.e., the expectation of a reward, or an empathy motive, i.e., the sharing of other’s emotions eliciting concern for the other’s welfare. Thus, motives and their interactions are not directly observable in overt behavior. Moreover, subjectively reported motives tend to be biased by social desirability (34).

Recent studies have started to use neuroscientific methods to distinguish different motives that result in the same behavior and to investigate multi-motive interactions (21, 22, 35–37). On a conceptual level, models of multi-motive interactions and their neural computation help to understand their impact on prosocial decisions. Practically, disentangling different motives and their interactions can shed light on the motivational basis of an observed behavior and inform interventions that foster or suppress certain behavioral outcomes. For example, differentiating between an egoistic and an empathy motive and/or investigating the interactions between both motivational forces can help improving incentive structures that shape individual prosocial behavior. The current review first provides a summary of the benefits and limitations of egoism- and empathy-based prosocial behaviors. Second, we review relevant neuroscience studies investigating the neural processing of egoism-based and empathy-based prosocial decisions, and the small number of studies that compared the neural processing of prosocial decisions elicited by the different motives. Third, we highlight future directions and open questions for future research.

## Advantages and disadvantages of egoism-based compared to empathy-based prosocial behavior

Before reflecting on potential differences between egoistically and empathically motivated prosocial behavior, a definition of both alternatives is necessary. *Egoism-based prosocial behavior* depends on the probability that prosocial behavior is associated with a reward (38–42), or the avoidance of costs (43). At the personal level, the pursuit of material goods is associated with lower psychological well-being both cross-sectionally and over time (44; for meta-analysis, see 45).

Although easily elicited, egoism-based prosocial behavior is fragile. There is evidence that incentives can undermine the behaviors that they were supposed to foster (46–50), known as crowding-out effects (51). One aspect of crowding-out relates to initial incentives vs. later prosocial behavior. In this context, it is proposed that crowding-out effects occur when a person receives a reward for a prosocial action and in consequence, is less likely to do the prosocial act for free later (52). Documenting the importance of crowding-out effects for real-life incentive structures, a large number of studies from behavioral economics (e.g., 53) have tested conditions that may alter the impact of incentives on prosocial behaviors. As another separate key outcome, they propose that incentives only undermine prosocial behaviors if they are offered in public (47, 53–55). If social interaction partners are aware that someone receives a payment that enhances prosocial behavior, this person loses their prosocial reputation (i.e., reputation loss, see Table 1). Consequently, this person is no longer perceived as a prosocial and nice person. For instance, Ariely et al. (47) asked participants to make donations to charitable organizations. These donations could be made publicly or privately and were incentivized or not. Incentives offered in private resulted in an increase in prosocial decision. In contrast, receiving payment for prosocial behavior in public reduced the prosocial decision frequency. Crowding-out effects have been observed in a variety of prosocial behaviors, including the collection of door to door money for charity (56), voluntary cooperation (57), and blood donations (58). According to other findings, the negative (i.e., decreasing) effect of reward on behavior is particularly strong when participants’ decisions are not anonymous and thus, their reputation is at stake. For instance, Newman and Cain (59) showed that individuals’ charitable behaviors were rated as more negative when they fulfilled self-interests compared to similar behaviors that did not result in a charitable benefit – a phenomenon they called “tainted altruism”. Other research confirmed reputational costs for egoistically motivated prosocial behavior (59–61).

**TABLE 1:** Summary of positive and negative aspects of egoism and empathy as motives of prosocial behavior.

	Egoistic motive		Empathy motive	
	Positive	Negative	Positive	Negative
	Easy to activate	Fragile	Long-lasting	Biased
Consequences of prosocial behavior for the actor	Resource gain	Reputation loss	Reputation gain	Resource loss

In summary, there may be a narrow and situation-specific region in public charitable behavior within which individuals can unleash their egoistic motive without fearing the loss of reputational benefits. This process may be dimensional rather than categorical, i.e., there is no general rule that indicates a tipping point at which egoism-based charity leads to reputational loss rather than gain.

The increase of own welfare is the most obvious aspect of an egoistic motive (i.e., resource gain, see Table 1), as incentivized prosocial behavior is rewarded by the value of the incentive and, depending on the context, can lead additionally to an increase in reputation. As an everyday example, in Germany a financial donation does not only support the work of a charity and increase the donor’s reputation. The donors also reduce their own tax burdens and thereby receive a financial advantage in return. Importantly, while in the case of egoism-based prosociality, a behavior that increases one’s own welfare also has a positive effect on others (e.g., monetary donations, paid blood/plasma donations), these benefits for others occur merely as a side effect but increasing one’s own welfare is the primary goal. The positive relationship between self-care and care for others has also been documented in recent research on egoistic motives (62) and the measurement of individual differences in trait egoism (63).

Traditionally, egoism has been linked to aggression (e.g., 3). However, under certain circumstances, even egoism-based aggression can be perceived as a prosocial act. For example, in a variety of models investigating social behavior, punishing norm deviating behavior is considered as a mechanism that facilitates the development of altruism, which makes the punishers to be perceived as altruists (64–66). A hitherto rarely addressed aspect is that egoistically motivated individuals may also punish other egoistic individuals in social interactions. By doing so, these individuals contribute to more prosocial behavior in general, although they ultimately aim to increase their chances of getting the maximum benefit by switching to an egoistic strategy at a later point. However, this kind of egoistic behavior could also occur in response to the presumably egoistic behavior of others, which is labeled “reactive egoism” (67). Egoists might fear exploitation by the egoistic behavior of others if they behave charitably, and are therefore motivated to behave in accordance with their own self-interest (e.g., 68, 69).

In contrast to egoistically-driven prosocial behavior, *empathy-based prosocial behavior* has the goal to increase the welfare of the other person (70). In more detail, although affective empathy results in two distinct reactions – compassion or personal distress (12, 71), only compassion is associated with empathy-based prosocial behavior. Therefore, while egoistically motivated behavior will stop in the absence of reward or distress, empathy-driven prosocial behavior based on compassion will continue until the welfare of the other is secured. There is a large body of evidence showing that empathic responses motivate cooperation and helping behavior (e.g., 25, 72). Practically, this means donating to charity, sharing with others, and volunteering time to help others (73).

However, acting on the basis of empathic feelings can also lead to less/a lack of prosocial behavior in general due to biases, as individual’s emotions can cloud their judgment regarding the best course of action (74). In the case of so called empathic vampirism (75), individuals experience the world vicariously through others and thus share their emotions, but without considering their interests. While this does not mean that empathic vampirism necessarily leads to disadvantageous effects, it is a possibility. An example for less or a lack of prosocial behavior is the identified victim effect. Experiments showed that participants donated more money for a the treatment of an ill child that was introduced with a picture and a name than to a non-identified child suffering from the same illness (76). Other empathy-induced biases include an increased empathy toward members of the empathizer’s group compared to members of different groups (77). Other research highlighted that the empathy toward the suffering of one particular person results in neglecting the fate of many other individuals, compared to the collective suffering of many individuals (e.g., 78). In this sense, empathy acts like a spotlight focusing on certain people or situations, thereby excluding others (79).

Besides biases leading to a reduction of prosocial behavior, sharing the emotions of others can also be disadvantageous for the empathizer, because it can elicit personal distress (80–83). Personal distress is a self-centered negative affect when being exposed to the suffering of others that may result in withdrawal instead of prosocial behavior toward the other. If withdrawal is not possible, personal distress may be the source of egoistically motivated prosociality, i.e., prosocial behavior with the goal to escape the distressing situation (i.e., to increase own welfare), instead of focusing on the welfare of the other person (24, 84–86). As an example, Piferi and colleagues (87) showed that personal distress was the most frequently reported motive for donations immediately following the terrorist attacks of September 11, whereas donations to alleviate the suffering of others was the most frequently cited motive one year after the attacks.

Taken together, both egoism and empathy have their bright and dark sides. Table 1 summarizes the positive and negative aspects of egoism and empathy as motives of prosocial behavior.

Based on the mere observation of overt behavior, empathic and egoistic motivation are hard to distinguish, because they can result in the identical behavior, i.e., the decision to help another person. There is evidence that neuroscience methods can help to specify the type of motive that drives a given decision. For example, Tusche and colleagues (37) identified empathy and perspective taking as motives for generous behavior and used functional magnetic resonance imaging (fMRI) to disentangle differential neural responses associated with each motive. Hein et al. (35) and Saulin et al. (22) showed that two different motives, i.e., empathy and reciprocity, incite a comparable frequency of prosocial decisions. However, based on the patterns of brain connectivity (35) and neural decision parameters (22), the authors were able to distinguish between the two motives and their interrelation. Moreover, in a recent meta-analysis using a data-driven graph-based approach, Rhoads and colleagues classified the neural circuitries of prosocial decisions based on task features. The results revealed three different clusters that were labeled as altruistic, cooperative or equity-based prosocial decisions. Compared to prosocial decisions driven by cooperation and equity, altruism-based prosocial decision uniquely recruited the dorsal anterior cingulate cortex (ACC) and the anterior insula (AI), i.e., regions that have been linked to the processing of empathy (20, 88, 89).

Inspired by these findings, the following section provides a concise review of the neural networks that have been related to the processing of egoism-related and empathy-related prosocial behavior and summarize the results of first studies that aimed to disentangle these motives based on these networks.

## Neural circuitries related to egoism-based prosocial decisions

The egoistic motivation of increasing one’s own outcomes and welfare (e.g., 25) has been linked to neural responses in reward circuitries (e.g., 90, 91). Human reward circuitries have been addressed in several seminal review articles (e.g., 92–95), showing that the regulation of behavioral and psychological responses to reward stimuli is coordinated by a series of cortical and subcortical structures (96). At the center of this circuit is the striatum, a subcortical structure involved in reward-related learning and resulting approach behaviors. Therefore, a variety of fMRI experiments have examined how the striatum with its potent connections to cortical regions and dopaminergic sites in the midbrain contributes to reward processing (e.g., 97–99).

In the context of prosocial decision behavior, the neural circuitries related to the processing of basic rewards have been linked to the intrinsic positive value of prosocial acts for the individual, known as “warm-glow.” For instance, in an early study Harbaugh et al. (100) asked participants to either observe money being transferred to a charity in a tax-like manner (mandatory condition) or voluntarily donated to the charity (voluntary condition). As a result, both mandatory and voluntary donations triggered similar neural activity in the ventral striatum (VS) as when participants received money themselves. Nevertheless, voluntary donation resulted in stronger striatal activation than mandatory donation, consistent with the concept of warm-glow giving. It was proposed that the act of giving to others itself is rewarding and results in a “warm-glow”, because it contributes to a positive self-image and a gain in reputation (55). Consequently, prosocial decisions that are driven by the expectation of a resulting “warm-glow” would be classified as egoistic, because they focus on a personal, intrinsic reward and an increase in reputation, instead of the welfare of the other (101–103).

Specifying these results, other studies using monetary donation task showed that the VS is part of a larger network, including the ventromedial prefrontal cortex (vmPFC; 104–106), as well as the junction between the temporal and the parietal cortex (TPJ; 107, 108). VS, vmPFC, and medial orbitofrontal cortex (mOFC) are associated with the computation of a common neural value currency for different types of goods, including social goods (95, 98, 109). Increases of activity within the TPJ during social decision-making has been linked to altruistic and generous decision-making (37, 107, 110), thereby promoting generosity. Moreover, in concert with the vmPFC, the TPJ has been related to overcoming egoistic impulses in prosocial decisions (e.g., 107, 108). For example, in an fMRI study by Strombach and colleagues, participants choose to keep a reward for themselves (egoistic decision) or divide this reward between themselves and a (close or socially distant) other person (prosocial decision; 91). An analysis of egoistic decisions alone revealed that vmPFC activity correlated with the value of the egoistic reward. However, when considering both egoistic and prosocial decisions, the results showed that an increase in prosocial decisions was associated with increased activation in the TPJ and the vmPFC. The individual strength of the connectivity between TPJ and vmPCF was larger for prosocial compared to egoistic decisions, an effect that was proposed to reflect the overcoming of egoistic impulses (111, 112). Recently, Sellitto et al. (113) tested framing effects on prosocial behavior in a social discounting task. Participants chose between egoistic and prosocial decisions either in a gain frame, i.e., prosocial decisions led to a reward for the other person, or in a loss frame, i.e., prosocial decisions prevented the other person from losing a previously received endowment. Supporting the results of other studies (e.g., 108), the authors demonstrated that activation in the TPJ and the vmPFC was linked to prosocial decisions in the gain frame, whereas insula activation was associated with prosocial behavior in the loss frame.

Another group of studies addressed how self-related reward-processing affects other motives. For example, Murayama et al. (50) studied whether a financial incentive undermines participants’ performance in a stopwatch task. In this task, participants were presented with an automatically starting stopwatch. The goal was to press a button to make the button press fall within 50 ms of the 5-s time point. In the first part of the study, participants received a performance-based reward, in the second part of the study this reward was no longer given. A control group completed the same task without performance-based reward in both periods. The results showed stronger activation in the striatum and midbrain in the reward group compared to the control group during the first part when the rewards were provided. In the second part of the study, this contrast was reversed, reflecting a decrease of striatal activation in the reward group compared to the control group in absence of an incentive. Based on this results, the authors concluded that the highly rewarding nature of money undermined an intrinsic motivation to successfully complete a task (see also 114). Although this research does not have a prosocial focus *per se*, it is important for us to understand how performance-based incentive systems practically guide human behavior.

Targeting neural activation and a potentially undermining effect of social reward (i.e., reputation effects) on monetary rewards in a social decision-making paradigm, Izuma et al. (115) asked participants to keep money for themselves or donate money to a charity while either being observed by others or not (control condition). At the behavioral level, participants donated more when they were observed by others. The neural results showed that activation in the striatum increased when participants made donations while being observed (compared to the absence of others), but also when they kept money for themselves without being observed (compared to the presence of others). As such, reputation effects increase the willingness to donate to a charity, which is encoded in striatal brain areas in a similar manner to egoistic decisions, such as keeping money (i.e., not donating).

## Neural circuitries related to empathy-based prosocial decisions

The association of brain regions with empathy-based prosocial behavior were mainly deduced from studies focusing on empathy-for-pain inductions as a manipulation for decision making. In these studies, participants are confronted with images or videos from persons or confederates suffering from pain compared to control conditions [for meta-analysis, see (11, 77, 116)]. The separation between cognitive empathy [or theory of mind (ToM)] and affective empathy is equally reflected at the neuronal level (9, 14, 15, 17, 117, 118). Cognitive empathy is commonly related to neural activation of the mPFC, the superior temporal sulcus (STS), the temporal poles (TP), and the TPJ (9, 13, 15, 16, 117). In contrast, affective empathy is usually linked to activity in the AI, ACC, and inferior frontal gyrus (IFG) (9, 13, 15, 16, 117, 119, 120). The relationship between different facets of empathy and prosocial decision-making has been reviewed in a large number of review articles and meta-analyses (121–124). Across these different previous reviews there is a broad agreement that neural activation in regions associated with affective empathy (e.g., AI, ACC) and cognitive empathy (e.g., TPJ, mPFC) are linked to empathy-based social decision-making in contrast to social decision-making without explicit activation of empathy for pain.

## Disentangling the neural circuitries related to the processing of egoistic and empathic motives

In an early study, Weiland et al. (125) aimed at disentangling the egoistic from the empathic motive in economic decision making, by comparing two different economic games, i.e., the ultimatum game (UG; where a proposer provides a monetary offer to a receiver who can decide to accept or reject the offer, but rejecting results in a payoff of zero for both), and the dictator game (where the allocator determines how to split an endowment and the receiver has no influence on the outcome). Weiland and colleagues assumed that fair offers in the ultimatum game are driven by strategic considerations with an underlying egoistic motive, and that fair offers in the dictator game are driven by an empathy motive. Within the UG, fair compared to unfair offers were associated with increased activation in the striatum, the superior temporal area and the temporal pole. In the dictator game, fair offers were associated with increased activity in the dorsal part of the ACC and the posterior cingulate cortex. Given that an increase in ACC activation has been associated with affective empathy (126, 127), the authors concluded that prosocial dictator giving might be driven by an empathy motive. However, as these results are based on the comparison of mechanistically different paradigms (i.e., UG and DG), they might exaggerate or overshadow the effects of the motivational states underlying the behavior and their neural correlates.

More recently, Cutler and Campbell-Meiklejohn (9) conducted an fMRI-based meta-analysis in which they synthesized findings from 36 studies focusing on prosocial giving (1,150 participants in total). The authors investigated the neural networks underlying “strategic” prosocial decisions (i.e., decisions that might increase the likelihood of extrinsic rewards) and “altruistic” decisions (i.e., decisions without the chance to receive extrinsic rewards). Strategic decisions included decisions based on the avoidance of punishment, reciprocity, rewards through cooperation or increased reputation, whereas altruistic decisions included decision driven by empathic concern or self-enhancement through compliance with social norms. According to the results, both altruistically motivated and strategically motivated prosocial decisions commonly recruit reward-related brain areas. Contrasting altruistically motivated and strategically motivated prosocial behaviors across these different paradigms revealed stronger activation in the ACC for decisions motivated by the predefined “altruistic” motives (including affective empathy) as compared to decisions motivated by “strategic,” egoistic motives. Overall, these results suggest that neural correlates can differentiate between giving to others based on egoistic motives and prosocial behavior without a chance to receive extrinsic rewards. However, the “altruistic” and “strategic” motive categories aggregated a number of very different motives. Thus, the neural differentiation between empathy-based and egoism-based remains rather unspecific. In addition, distinct types of decisions might also pertain to both the “altruistic” and the “strategic” category. For instance, self-enhancement through compliance with social norms could also categorized as “strategic” because decisions in domain may later entail positive consequences for the agent.

Disentangling egoism-based and empathy-based prosocial behavior requires paradigms that allow for investigating the effect of both motives on the same behavior. In a recent study, Iotzov and colleagues induced empathy for pain and egoism and investigated the effect of both motives on an identical social decision task (21). In one condition (empathy-alone condition) participants observed their partner experiencing pain. In the other condition (empathy-bonus condition), participants also observed the other in pain, but were offered a financial bonus if they made prosocial decisions in the majority of trials in a subsequent task, in which they could allocate points in favor of themselves or in favor of the other person. Thus, in the empathy-bonus condition, the egoistic motive of outcome maximization (winning the bonus) was activated simultaneously with the empathy motive. Later, participants performed the same social decision task in which they could allocate points in favor of themselves or in favor of the other person. The results showed more prosocial decisions in the empathy-bonus compared to the empathy-alone condition. Clarifying the mechanism through which the bonus facilitated empathy-based decisions, drift-diffusion modeling (DDM) revealed an increase in the efficiency of information processing (drift rate). On the neural level, the bonus-related increase in drift rate was captured by changes of neural responses in the AI, i.e., the same region that also correlated with empathy ratings. Interestingly, the effect of the bonus on empathy-related neural responses in AI was stronger the lower an individual scored on empathy. In contrast, in highly empathic individuals the bonus had little effect.

**FIGURE 1:**
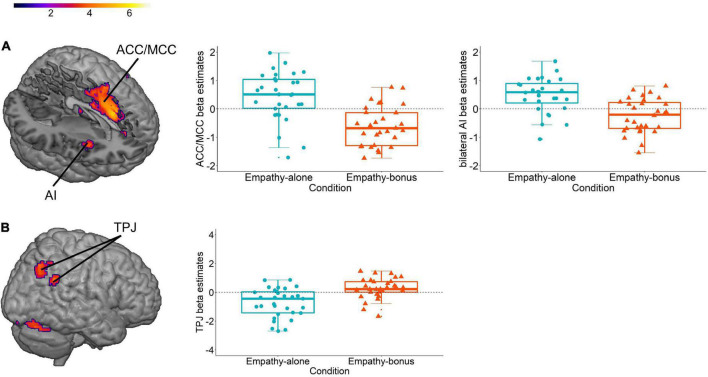
Neural response during prosocial decisions (1% uncorrected cluster-based inference with *p* < 0.001 cluster-forming threshold; *k* = 0). We used data (*N* = 31) from Iotzov et al. (21) and reanalyzed them for the purpose of this review. **(A)** Neural contrast between prosocial decisions in the empathy-alone vs the empathy-bonus condition and visualization of the average beta-values extracted from the clusters of activation in the bilateral anterior insula (AI; center co-ordinates left *x* = –36, *y* = 14, *z* = 8 and right *X* = 30, *y* = 11, *z* = –16) and the anterior and mid cingulate cortex (ACC/MCC; center co-ordinates *x* = –9, *y* = 14, *z* = 35). **(B)** Neural contrast between prosocial decisions in the empathy-bonus vs the empathy-alone condition and visualization of the average beta-values extracted from two clusters of activation in the temporal parietal junction (TPJ; center co-ordinates *x* = –45, *y* = –55, *z* = 32 and *x* = –36, *y* = –70, *z* = 47).

Comparing the neural network involved in prosocial decisions in the empathy-alone compared to the empathy-bonus condition revealed activations in the ACC and midcingulate cortex (MCC), as well as the AI (Figure 1A), the neural network that has been associated with the processing of affective empathy for pain (11, for meta-analyses, see 120, 128). The reverse contrast (empathy-bonus vs empathy-alone) revealed activation in the TPJ (Figure 1B), a brain region that has been associated with cognitive empathy or theory of mind (129, 130) and overcoming egoistic impulses (107, 108). Together, these findings imply that an egoistic motive (incited by a monetary incentive) alters the cognitive and neural processing of empathy-based prosocial decisions. Moreover, they indicate different neural networks for the processing of egoism-based and empathy-based prosocial behavior, in line with other recent evidence (e.g., 9).

## Summary and perspectives

In the first part of our review, we reviewed behavioral evidence for egoism-based prosocial behavior, compared to prosocial behaviors driven by empathy. We conclude that egoism can be a strong driver of prosocial behaviors, that is however limited in several aspects. First, compared to empathy-based prosocial behavior, egoism-based prosocial behavior focuses on increasing one’s own well-being, whereas increasing the well-being of the other person plays only a subordinate role and can be seen more as a means to an end. Second, the two motives differ in their effects on reputation. While empathy-based prosocial behavior has a positive effect on reputation, adding an egoistic motive can lead to a reduction in reputation (47, 53–55).

That being said, egoism and empathy can incite the same behavioral outcomes, e.g., prosocial decisions. Thus, it can be difficult to infer an egoistic motivation from overt behavior. If one only focuses on the behavioral outcome *per se*, it may not seem necessary to specify the motivational source of the behavior, i.e., to draw a distinction between an egoistic and another motive. However, differentiating between different motives is crucial for behavioral predictions. Given that it is conditional on reward, egoistically motivated prosocial behavior will stop if this reward is not provided. In contrast, prosocial behavior driven by empathy should continue until the wellbeing of the other person is secured.

In the second part of the review, we asked if neuroscientific evidence can help to specify the motive that drives a given behavior, for example, by specifying if a prosocial decision is driven by an egoistic or an empathic motive. The reviewed neural evidence indeed suggests differential neural circuitries for the processing of egoism-based and empathy-based prosocial decisions.

However, neuroscientific studies that directly compare egoism-based prosocial behavior with prosocial behavior that is driven by a specific other motive are rare. Moreover, they are mostly based on the comparison between different paradigms (e.g., DG vs. UG), one of which is assumed to promote egoistic decisions and the other is assumed to promote “altruistic” decisions (9, 125). Based on these approaches it remains unclear whether potential neural differences reflect differences in motivational states or in task requirements. One possible solution to this challenge is to design carefully controlled experiments in which individuals perform the same prosocial task driven by egoism and driven by another motive see (e.g., 21). These paradigms should be combined with state-of-the-art neuroscientific methods to assess changes in neural activation and neural connectivity in complex motivational states (i.e., states characterized by different interacting motives) and simple motivational states (in which one particular motive prevails). Moreover, computational methods should be used to specify the effect of interacting motives on the decision process. For example, first studies have started to use drift-diffusion modeling (131, 132) to determine how different motivational states alter the different components of the decision process (i.e., influence the initial decision bias or the efficiency of information accumulation (21, 133). As another line of future research, it would be interesting to investigate how “state”-egoism, i.e., egoism that is induced by incentives, interacts with trait egoism, and how both forms of egoism shape other specific motives.

## Author contributions

MW and GH: conceptualization and writing – original draft. GH: supervision. MW and VI: visualization. MW, VI, YZ, and GH: writing – review and editing. All authors contributed to the article and approved the submitted version.
